# Knowledge and practices of dietary iron and anemia among early adolescents in a rural district in Ghana

**DOI:** 10.1002/fsn3.2249

**Published:** 2021-04-02

**Authors:** Michael Akenteng Wiafe, Charles Apprey, Reginald Adjetey Annan

**Affiliations:** ^1^ Department of Biochemistry and Biotechnology Kwame Nkrumah University of Science and Technology Ashanti Ghana

**Keywords:** adolescents, anemia, dietary iron, knowledge, practices

## Abstract

The study assessed knowledge and practices of dietary iron intake and anemia among early adolescents in the Asante‐Akim Municipality of Ghana. A cross‐sectional study was conducted among 137 adolescents, aged 10–14 years. Structured questionnaire was used to collect data on sociodemographic, knowledge of iron, and anemia, and iron intake practices. Hemoglobin levels were determined using Hemocue 301. Data were analyzed using descriptive, chi‐square test, and binary logistics regression. The mean age of participants was 11.5 years. About 40% had knowledge of iron deficiency anemia, 29.4% knew anemia causes, 86% knew symptoms of anemia, and 35% knew anemia consequences. Although 41.2% of participants knew its prevention as eating iron‐rich foods, 31.4% knew the food sources of iron, and 4.4% mentioned animal‐based foods as rich sources, with the bulk (27%) mentioning plant‐based foods instead. Moreover, 18.2% knew iron enhancers, while 0.7% knew iron inhibitors. More chicken consumers (72.2%) than nonconsumers (56.6%) met the Estimated Average Requirement for dietary iron intake (Chi‐square 3.4, *p* = .073), while more dried fish consumers (88%) than nonconsumers (66.7%) had normal hemoglobin levels (Chi‐square 4.5, *p* = .050). Knowledge of food sources of iron and iron‐rich foods was positively associated with intake of chicken, fresh fish, and dried fish. Moreover, lower knowledge of iron‐based food sources (β = −1.015, *p* = .020) and iron‐rich foods (β = −2.188, *p* = .015) was inversely associated with beef intake. Anemia was negatively associated with chicken (β = −0.310, *p* = .416) and dried fish (β = −1.299, *p* = .045) consumption. Majority of the adolescents had low knowledge of iron. Chicken and dried fish intake reduced the risk of anemia development. Our study may be the first to assess knowledge on iron, anemia, and its impact among young Ghanaian adolescents. Our findings provide insights into this topic, calling for to improve knowledge, and practices on anemia in Ghanaian adolescents.

## Graphical abstract

Anaemia, a type of micronutrient deficiency, is known to affect the physical and cognitive abilities of adolescents. The study assessed knowledge and practices of dietary iron intake and anaemia. Results show that majority of adolescents had little knowledge of iron, causes of anaemia and sources of iron‐rich foods.

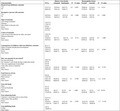

## 1 INTRODUCTION

Early adolescence, which begins the adolescence phase, lasts for five years and expands from age 10 to 14 years old (Kinghorn et al., 2018; Sawyer et al., 2018). Rapid physiological, sexual, and neurological changes in adolescent growth require adequate intake of energy, protein, iron, zinc, and other micronutrients (Das et al., 2017; Patton et al., 2016). Iron improves cognitive function and physical performance in adolescents (Blanton, 2014; Mousa et al., 2016; Scott et al., 2018).

Inadequate intake of dietary iron and iron‐enhancing foods, among other factors, contributes to anemia in most developing countries (Geissler & Singh, 2011; Kaur, 2016.). According to the Ghana Demographic and Health Survey (2014), anemia mostly occurs due to malaria, helminthic infections, and micronutrient deficiency such as iron, vitamin A, vitamin B12, folate, and zinc.

The widespread of iron deficiency anemia among adolescents in different geographical settings has been reported in several studies (Chandrakumari et al., 2019; Hassan et al., 2017; Mistry et al., 2017; Shaka & Wondimagegne, 2018). Iron deficiency anemia has been prioritized as a major nutritional burden of adolescents across the globe (World Health Organization, 2011). In Africa however, anemia is more prevalent in young adolescents and young women in Ghana, Benin, and Mali (Yasutake et al., 2013).

Adolescents diagnosed with iron deficiency anemia may suffer from its negative effect including but not limited to physical and psychiatric disorders. Anemia is associated with an increased risk of psychiatric disorders such as attention‐deficit hyperactive disorders, unipolar and bipolar depression disorders, delayed mental development, and mental retardation (Chen et al., 2013). Iron deficiency anemia also contributes to about 50% of lives lost to disability in adolescents annually (Lassi et al., 2017).

Siddiqui et al., (2013), in their study, indicated that adolescents had acceptable knowledge levels about the causes, treatment, and prevention of anemia. However, several studies in other geographical locations also concluded that adolescents showed nutrition knowledge deficit of iron and causes, consequences, management, and prevention of anemia (Jalambo et al., 2017; Kakkar et al., 2019; Melwani et al., 2018; Pareek & Hafiz, 2015; Singh et al., 2019). These studies infer that most adolescents have low nutritional knowledge of anemia. Thus, knowledge of anemia is key in management and prevention.

Awareness of anemia and its relationship with iron‐rich food intake was known by very few female adolescents in Ethiopia and India (Gebreyesus et al., 2019; Latha & Mohan, 2017). Most adolescent girls with average‐to‐good knowledge levels of anemia and its prevention showed high levels of anemia (Ahwal, 2016). This might suggest that knowledge of anemia does not necessarily help maintain or increase hemoglobin levels.

Few studies have reported high nutrition knowledge related to iron, iodine, vitamin A, and their consequences (Latha & Mohan, 2017; Melaku et al., 2018; Spronk et al., 2015). High nutrition knowledge positively contributed to high intake of fruits, vegetables, and carbohydrate‐rich foods (Alaunyte et al., 2015; Asakura et al., 2017; Spronk et al., 2015). Leonard et al., (2014), also indicated that knowledge about iron food sources was positively related to dietary iron intake. Dietary practices had a significant positive relationship with hemoglobin levels (Shahzad et al., 2017). Consumption of iron‐rich foods and vitamin C‐rich foods showed increased bioavailability of iron (Lane et al., 2016). Withdrawal of iron‐fortified foods among male adolescents with frequent consumption of meat and vitamin C did not alter the rate of iron deficiency (Sjöberg & Hulthén, 2015). A systematic review showed that most studies found positive association between nutrition knowledge and dietary intake (Heaney et al., 2011).

Ronto et al., (2016) found that although adolescents appreciate that knowledge on food and nutrition is essential for good dietary behavior, they do practice otherwise. In Malaysia, low consumption of iron‐rich foods was observed among anemic and nonanemic adolescent girls (Kapu et al., 2019). A study in India noted inadequate dietary iron intake among adolescent boys (Shafiee et al., 2015). Female adolescents with anemia also showed inadequate dietary iron intake (Mazhar, 2015). Poor dietary iron intake was reported among adolescent girls showing satisfactory knowledge level of iron‐rich foods (Siddiqui et al., 2013). A summary of systematic review indicates that anemia among adolescents in Africa is largely due to inadequate dietary iron intake (Harika et al., 2015, 2017). Scientific research admits gaps between knowledge and practices of dietary iron intake among adolescents.

In view of the knowledge deficit on anemia and inadequate intake of dietary iron among adolescents in other jurisdictions, the current study aimed to assess knowledge and practices of dietary iron intake and anemia among early adolescents in a rural district in Ghana.

## 2 MATERIALS AND METHODS

### 2.1 Study population and design

The study was conducted in Asante‐Akim South Municipal, a rural district in Ghana. A total of one hundred and thirty‐seven (137) early adolescents (10–14 years old) were recruited in this cross‐sectional study. Participants and guardians who gave their consent qualified for the study.

### 2.2 Questionnaire

Structured questionnaires, adapted from Food and Agriculture Organization (FAO) document titled “Guidelines for assessing nutrition‐related knowledge, attitudes and practices’ (Food & Agriculture Organization, 2014), were used to assess knowledge of iron and food intake or nutrition practices.

### 2.3 Assessing the knowledge level of Iron

Seven questions adapted from the FAO document were used to assess the knowledge level of iron. The questionnaire was categorized into: a) awareness and general signs of iron deficiency anemia; b) causes of iron deficiency anemia; c) consequences of iron deficiency anemia for young children; d) prevention of iron deficiency anemia; e) iron‐rich foods; f) foods that increase iron absorption; and g) foods that decrease iron absorption.

### 2.4 Nutrition practices

Structured questionnaires for assessing nutrition practices were also adapted from the FAO document and put under three headings: a) Practices that increase intake of iron‐rich foods, b) consumption of vitamin C‐rich foods, and c) consumption of coffee/tea. Iron‐rich foods consumed in the past 24‐hr were classified into organ meat, flesh meat, insects, and fish and seafood. Furthermore, multiple past 24‐hr recall was used to determine dietary iron intake. Adequate dietary iron intake was defined as 8mg/day as recommended by the National Academy of Sciences (Otten et al., 2006).

### 2.5 Hemoglobin determination

HemoCue Hb 301 analyzer (HemoCue AB, SE‐262 23, Ӓngelholm, Sweden) was used to analyze hemoglobin (Hb) levels of participants on the field. The pointed tip of the microcuvette was placed into the blood drop. The microcuvette was filled automatically with ten microliters (10μL) of the blood by capillary action. Excess blood on the cuvette was cleaned with lint‐free wipe. The microcuvette was inspected for air bubbles. The open end of the microcuvette was not touched. The microcuvette was placed in the holder of the hemoCue and was gently pushed into the photometer, and the results were displaced by approximately ten (10) seconds. The hemoglobin was recorded. Anemia was defined as Hb less than 11g/dl as given by the manufacturer's manual.

### 2.6 Statistical analysis

Statistical Package for Social Sciences (SPSS version 25) was used for data cleaning and analysis. Descriptive statistics were used to analyze the sociodemographic characteristics and knowledge level of: anemia, sources of iron, iron‐rich foods, iron‐enhancing foods, and iron‐inhibiting foods. Chi‐square test was done to assess the relationship between knowledge level of iron, food intake practices, iron intake, and hemoglobin status. Data were presented as mean, standard deviation, frequency, and percentages. Binary logistics regression was used to predict the relationship between dietary iron intakes such as beef, chicken, fresh fish, dried fish, and knowledge of sources of iron, iron‐rich foods, and hemoglobin status. A *p* < .05 was considered statistically significant.

## 3 RESULTS

One hundred and thirty‐seven adolescents took part in the study. Participants had a mean age of 11.5 ± 1.1 years. About 70.1% and 29.9% had primary school and Junior High School (JHS) education, respectively. Of the 50.4% of male participants, 75.4% had primary education and 24.6% attended JHS. Meanwhile, 64.7% and 35.3% of the female adolescents had primary and JHS education, respectively. The guardians were predominantly farmers (56.9%) followed by traders (28.5%), others (13.9%), and teachers (0.7%). Most of the guardians fell within the low‐ (73%) and medium (26.3%)‐ income categories (Table 1).

**TABLE 1:** Sociodemographic characteristics of participants

Variable	*N* (%)	Mean ± *SD*	Level of education	
			Primary	JHS
Age				
Male	69 (50.4)	11.4 ± 1.1	52 (75.4)	17 (24.6)
Female	68 (49.6)	11.6 ± 1.1	44 (64.7)	24 (35.3)
Total	137	11.5 ± 1.1	96 (70.1)	41 (29.9)
Guardians occupation				
Teacher	1 (0.7)			
Farmer	78 (56.9)			
Trader	39 (28.5)			
Others (cleaner, seamstress)	19 (13.9)			
Income status				
Low	100 (73.0)			
Medium	36 (26.3)			
High	1 (0.7)			

Table 2 represents hemoglobin status by study participants. More males (60%) were anemic compared to females (40%). Participants with primary education (77.5%) had higher prevalence of anemia relative to those with JHS education (22.5%).

**TABLE 2:** Hemoglobin status by study participants

Variable	Hemoglobin status			
	Nonanemia	Anemia	χ^2^	*p*‐value
Gender				
Female	51 (53.1)	16( 40.0)	1.9	.190
Male	45 (46.9)	24 (60.0)		
Community				
A	48 (54.2)	20 (50)	2.3	.187
B	48 (45.8)	20 (50)		
Level of education				
Primary	65 (67.7)	31 (77.5)	1.3	.305
JHS	31 (32.3)	9 (22.5)		
Iron supplement				
Yes	81 (84.4)	32 (80.0)	0.4	.617
No	15 (15.6)	8 (20.0)		
Whom do you live with?				
Both Parents	64 (66.7)	23 (57.5)	2.3	.311
Single Parent	17 (17.7)	12 (30.0)		
Nonparent	15 (15.6)	5 (12.5)		
Frequency of Deworming				
Regularly	6 (6.2)	4 (10.0)	0.6	.480
Nonregularly	90 (93.8)	36 (90)		
Menstruating				
Yes	10 (19.6)	1 (6.2)	1.6	.274
No	41 (80.4)	15 (93.8)		

Note

Chi‐square test (χ^2^); Fisher's Exact test, *p* <.05.

Table 3 presents knowledge level, dietary iron intake, and hemoglobin status. Chi‐square showed no statistically significant (*p* > .05) relationship between participants’ knowledge level, dietary iron intake, and hemoglobin status. About 37.2% and 62.8% responded “yes” and “no,” respectively, to the awareness of iron deficiency anemia. In the anemia group, 30% of participants had heard of iron deficiency anemia and 70% had not. Among participants who were aware of iron deficiency anemia, 70.6% responded “yes” and 29.4% “no” to identifying persons with anemia. Nearly, 86% of participants knew the signs of anemia. Majority of the participants responded “don't know” as against “know” about the: causes of anemia (70.6% versus 29.4%), consequences of anemia in children (64.7% versus 35.3%), prevention of anemia (58.8% versus 41.2%), sources of iron (68.6% versus 31.4%), iron‐rich foods (95.6% versus 4.4%), iron ‐enhancing foods (81.8% versus 18.2%), and iron‐inhibiting foods (99.3% versus 0.7%).

**TABLE 3:** Knowledge level, dietary iron intake, and hemoglobin status

Knowledge of anemia and iron	*N*(%)	Dietary iron intake				Hemoglobin status			
		Adequate	Inadequate	χ^2^	P‐value	Normal	Anemic	χ^2^	P‐value
Heard of iron deficiency anemia									
Yes	51 (37.2)	33 (38.4)	18 (35.3)	0.1	0.855	39 (40.6)	12 (30.0)	1.3	0.331
No	86 (62.8)	53 (61.6)	33 (64.7)	57 (59.4)	28 (70.0)				
Recognize a person with anemia									
Yes	36 (70.6)	23 (69.7)	13 (72.2)	0.0	1.000	27 (69.2)	9 (75.0)	0.1	1.000
No	15 (29.4)	10 (30.3)	5 (27.8)	12 (30.8)	3 (25.0)				
Signs of anemia									
Less energy/weakness	12 (33.3)								
Paleness	17 (47.2)								
More likely to become sick	2 (5.6)								
Know	31 (86.1)								
Don't know	5 (13.9)								
Causes of anemia									
Lack of iron in the diet/eat too little, not much	12 (23.5)								
Sickness/infections	3 (5.9)								
Know	15 (29.4)	9 (27.3)	6 (33.3)	0.2	0.751	12 (30.8)	3 (25.0)	0.1	1.000
Don't know	36 (70.6)	24 (72.7)	12 (66.7)	27 (69.2)	9 (75.0)				
Consequences of children with iron deficiency anemia									
Delay of mental and physical development	8 (15.7)								
Other (death)	10 (19.6)								
Know	18 (35.3)	10 (30.3)	8 (44.4)	1.0	0.367	15 (38.5)	3 (25.0)	0.7	0.502
Don't know	33 (64.7)	23 (69.7)	10 (55.6)	24 (61.5)	9 (75.0)				
How can anemia be prevented?									
Eat iron‐rich foods	14 (27.4)								
Eat Vitamin C‐rich foods during or right after meals	3 (5.9)								
Take iron supplements if prescribed	3 (5.9)								
Treat other causes of anemia	1 (2.0)								
Know	21 (41.2)	12 (36.4)	9 (50.0)	0.9	0.385	17 (43.6)	4 (33.3)	0.4	0.739
Don't know	30 (58.8)	21 (63.6)	9 (50.0)	22 (56.4)	8 (66.7)				
Food Sources of iron									
Animals sources	6 (4.4)								
Plant sources	37 (27.0)								
Know	43 (31.4)	26 (30.2)	17 (33.3)	0.1	0.708	34 (35.4)	9 (22.5)	2.2	0.161
Don't know	94 (68.6)	60 (69.8)	34 (66.7)	62 (64.6)	31 (77.5)				
Iron‐rich foods									
Meat	2 (1.5)								
Fish and Seafoods	4 (2.9)								
Know	6 (4.4)	4 (4.7)	2 (3.9)	0.0	1.000	5 (5.2)	1 (2.5)	0.5	0.670
Don't know	131 (95.6)	82 (95.3)	49 (96.1)	91 (94.8)	39 (97.5)				
Iron‐enhancing foods									
Vitamin C‐rich foods such as citrus fruits									
Other (meat, fish, and poultry)	21 (15.3)								
Know	4 (2.9)								
Don't know	25 (18.2)	14 (16.3)	11 (21.6)	0.6	0.495	21 (21.9)	4 (10.0)	2.7	0.145
Iron‐inhibiting foods	112 (81.8)	72 (83.7)	40 (78.4)	75 (78.1)	36 (90.0)				
Coffee	1 (0.7)								
Don't know	136 (99.3)								

Note

Chi‐square test (χ^2^); Fisher's Exact test, *p* <.05; animal sources **(**meat, fish, and crabs); plant sources (Turkey berry, cocoyam leaves).

Food intake practices, iron intake, and hemoglobin status are presented in Table 4. The study showed statistically insignificant (*p* > .05) relationship among food intake practices, iron intake, and hemoglobin status.

**TABLE 4:** Food intake practices, iron intake, and hemoglobin status

Food intake	Iron intake				Hemoglobin status			
	Up to EAR	<EAR	χ^2^	*p*‐value	Normal	Anemia	χ^2^	*p*‐value
Beef								
Yes	20 (23.3)	8 (15.7)	1.1	0.382	22 (22.9)	6 (15.0)	1.1	0.358
No	66 (76.7)	43 (84.3)			74 (77.1)	34 (85.0)		
Chicken								
Yes	39 (45.3)	15 (29.4)	3.4	0.073	36 (37.5)	18 (45.0)	0.7	0.446
No	47 (54.7)	36 (70.6)			60 (62.5)	22 (55.0)		
Fresh fish								
Yes	44 (51.2)	29 (56.9)	0.4	0.596	50 (52.1)	23 (57.5)	0.3	0.578
No	42 (48.8)	22 (43.1)			46 (47.9)	17 (42.5)		
Dried fish								
Yes	17 (19.8)	8 (15.7)	0.4	0.650	22 (22.9)	3 (7.5)	4.5	0.050
No	69 (80.2)	43 (84.3)			74 (77.1)	37 (92.5)		
Citrus fruits								
Yes	83 (96.5)	46 (90.2)	2.3	0.148	90 (93.8)	38 (95.0)	0.1	1.000
No	3 (3.5)	5 (9.8)			6 (6.2)	2 (5.0)		
Coffee or Tea								
Yes	49 (57.0)	22 (43.1)	2.5	0.157	51 (53.1)	20 (50.0)	0.1	0.851
No	37 (43.0)	29 (56.9)			45 (46.9)	20 (50.0)		

Note

Chi‐square; *p* <.05;

Abbreviation: EAR, Estimated Average Requirement.

NB: Insect (Insect larvae, red ants, grasshoppers, crickets, termites), flesh meat (pork, dog, and rabbit), prawns, and shrimps were not consumed for the past 24hours. Canned fish, duck, lamb, goat, and organ meat (liver, kidney, and heart) were negligibly consumed for the past 24hours.

Table 5 represents binary logistics regression of knowledge, practices of dietary iron, and anemia. The results showed that knowledge about sources of iron was positively associated with consumption of chicken (β = 0.280, *p* = .275), fresh fish (β = 0.396, *p* = .284), and dried fish (β = 0.197, *p* = .687) consumption, respectively. Having knowledge on iron‐rich foods was positively associated with dietary intake of chicken (β = 0.275, *p* = .756), fresh fish (β = 0.861, *p* = .330), and dried fish (β = 18.331, *p* = .998). However, participants with knowledge of sources of iron (β= −1.015, *p* = .020) and iron‐rich foods (β= −2.188, *p* = .015) had statistically significant and inverse association with beef intake. Dietary intake of chicken (OR = 0.7, *p* = .416, 95%CI = 0.3–1.5) and dried fish (OR = 0.2, *p* = .045, 95%CI = 0.1–1.0) reduced the prevalence of anemia. Conversely, the intake of beef (OR = 1.7, *p* = .302, 95%CI = 0.6–4.5) and fresh fish (OR: 1.2, *p* = .564, 95%CI = 0.6–2.6) increased the risk of developing anemia.

**TABLE 5:** Binary regression of knowledge, practices of dietary iron, and anemia

Dietary Intake	Food Sources of Iron (Know)		Iron‐Rich Food (Know)		Anemia		
	β	*p*‐value	β	*p*‐value	β	OR(95%CI)	*p*‐value
Beef							
Yes	−1.015	0.020	−2.188	0.015	0.522	1.7 (0.6–4.5)	.302
No						1	
Chicken							
Yes	0.280	0.463	0.275	0.756	−0.310	0.7 (0.3–1.5)	.416
No						1	
Fresh Fish							
Yes	0.396	0.284	0.861	0.330	0.219	1.2 (0.6–2.6)	.564
No						1	
Dried Fish							
Yes	0.197	0.687	18.331	0.998	−1.299	0.2 (0.1–1.0)	**.045**
No						1	

Note

Binary logistics regression; *p* < .05.

## 4 DISCUSSION

The study aimed at assessing the knowledge and practices of dietary iron intake and anemia of early adolescents in a rural district in Ghana. Findings from this study showed that majority of the adolescents were not aware of iron deficiency anemia and had less knowledge about the causes, consequences on children's health, and prevention. Food sources of iron, iron‐rich foods, and foods that aid or reduce iron absorption were known by very few participants. Knowledge about food sources of iron and iron‐rich foods was positively associated with intake of chicken, fresh fish, and dried fish, although a statistically significant and inverse association was observed with beef. Consumption of fresh fish and beef increased the risk of anemia development, and chicken and dried fish showed otherwise. Dried fish intake significantly reduced the occurrence of anemia.

Our study showed that most of the adolescents (62.8%) were not aware of iron deficiency anemia. However, it was lower (88.9%) than that reported by Srinivas and Mankeshwar, (2015). Lower anemia knowledge (34.9%) than the present study has been documented by Singh et al., (2019). It suggests that many adolescents have anemia knowledge deficit. We also observed that most of the participants who were unaware of anemia were also victims of anemia. This finding is analogous with Gebreyesus et al., (2019), study conducted in Ethiopia. Majority of the adolescents also had limited knowledge about the causes, consequences, and prevention of anemia. The outcome was consistent with other studies conducted in India among adolescents (Kakkar et al., 2019; Melwani et al., 2018).

In this study, approximately 95.6% of the adolescents did not know about foods rich in iron, and this outcome concurred with a study conducted in Palestine, which indicated that about 90% of the adolescents had very little knowledge about iron‐rich foods (Jalambo et al., 2017). In India, Latha and Mohan et al., (2017) showed that majority of adolescents had higher knowledge about iron‐rich foods contrary to our study outcome. Most of the participants in our study had primary education which may have accounted for the low knowledge level. The few who had knowledge of iron‐rich foods mostly mentioned fish and seafood, with meat being the least. Majority of the participants did not know about foods that increase or reduce iron absorption, although the outcome was slightly higher than that reported by Kakkar et al., (2019). This might suggest that most adolescents are not interested in reading food labels. A greater proportion of the participants indicated that cocoyam leaves (“*Kontomire*”) and turkey berries (“*Kwahu nsusua”*) were the main sources of iron as reported by Omari et al., (2017) findings in rural communities in Ghana. Perhaps, health educators and guardians might be promoting these foods as iron‐rich in most rural communities.

The current study indicated that participants with high knowledge about food sources of iron and iron‐rich foods also had high intake of chicken, fresh fish, and dried fish, with statistically insignificant association. Our outcome was similar to Leonard et al., (2014), who found positive relationship between food sources of iron and dietary iron intake. Higher nutrition knowledge was positively associated with higher dietary intake particularly with fruits and vegetables (Asakura et al., 2017; Heaney et al., 2011; Spronk et al., 2015). However, in the present study, beef consumption was statistically significant and inversely associated with participants’ nutrition know‐how of iron‐rich foods and food sources of iron. Participants’ knowledge about animal sources of food was very limited as it was evident that none consumed insects, flesh meat, prawns, and shrimps with a negligible intake of duck, lamb, goat, organ meat, and canned fish. The absence of consumption of some iron‐rich foods especially insects may be that they were not in season and unavailable (Manditsera et al., 2018). In addition, insects used as food sources are mostly consumed in the Northern part of Ghana with the exception of palm weevil (Anankware et al., 2016). Also, most of the adolescents were likely to eat out of home and may have limited purchasing power to afford them as it was demonstrated that majority of guardians fell in the low‐income group. Adolescents with families of low socioeconomic status had reduced intake of iron‐rich foods (Shafiee et al., 2015).

Anemia occurrence was negatively associated with chicken and dried fish intake which means consumption of these foods could reduce the risk of anemia. In this study, participants frequently consumed fish and poultry other than liver, kidney, and heart. Even though iron is largely distributed in the liver, kidney, and heart of animals (meat and poultry), consumption of the right amount of other parts of the animal could also contribute to meeting the iron requirement. This was expected as meat, poultry, and fish contain intrinsic factor and intake of vitamin C‐rich foods enhances iron absorption (Lane et al., 2016; Lopez et al., 2016). This outcome concurred with another study in Japan (Imai & Nakade, 2019). In our study, meat, fish, and crabs were often mentioned as animal sources of food as it may be due to their availability, affordability, and frequent consumption in the community.

## 5 CONCLUSION

Participants had low knowledge of iron deficiency anemia, causes, consequences, and prevention. Few adolescents had knowledge of food sources of iron, iron‐rich, iron‐enhancing, and iron‐inhibiting foods. Participants showed no consumption of insects, prawns, shrimps, seafood, pork, rabbit, dog and very low intake of liver, kidney, and heart. Knowledge of food sources of iron and iron‐rich foods improved intake of chicken, fresh, and dried fish except for beef. Dried fish and chicken intake reduced the occurrence of anemia. Stakeholders in promoting adolescent health should intensify nutrition education on iron.

## CONFLICT OF INTEREST

The authors declare that they have no conflicts of interest.

## AUTHOR CONTRIBUTIONS

Michael Akenteng Wiafe involved in conceptualization—supporting, formal analysis—supporting, investigation—equal, project administration—equal, writing original draft—equal. Charles Apprey involved in data curation—equal, formal analysis—lead, supervision—supporting, visualization—lead, writing, review and editing—lead. Reginald Adjetey Annan involved in conceptualization—lead, formal analysis—lead, investigation—lead, project administration—lead, supervision—lead, writing, review and editing—lead.

## ETHICAL APPROVAL

The study protocol was approved by the Department of Biochemistry and Biotechnology, and ethics approval was given by the Committee on Human Research, Publication and Ethics at the Kwame Nkrumah University of Science and Technology, Ghana. Consent of guardians and participants was obtained before recruitment.

## Data Availability

Data used to support the findings of this study may be assessed by writing to the Chairman Committee on Human Research Publication Ethics, Room 8 Anatomy Block 3, School of Medical Sciences, Kwame Nkrumah University of Science and Technology, Kumasi, Ghana, or chrpe.knust.kath@gmail.com.
